# Molecular genetic and pregnancy outcomes of fetuses with increased Nuchal Translucency

**DOI:** 10.1371/journal.pone.0354363

**Published:** 2026-08-07

**Authors:** Luoyuan Cao, Wenxu Dong, Xiaomin Huang, Qinjuan Wu, Jing Yang, Jiaojiao Lu, Pengsheng Lin, Xunyan Chen, Xian Zheng, Xiaomei Zeng, Xianguo Fu

**Affiliations:** 1 Ningde Clinical Medical College of Fujian Medical University, Ningde, People’s Republic of China; 2 Department of Central Laboratory, Ningde Municipal Hospital, Ningde, Fujian, People’s Republic of China; 3 Department of Ultrasound, Ningde Municipal Hospital, Ningde, Fujian, People’s Republic of China; 4 Department of Obstetrics, Ningde Municipal Hospital, Ningde, Fujian, People’s Republic of China; 5 Public Health School of Fujian Medical University, Fuzhou, Fujian, People’s Republic of China; Shaheed Rajaei Cardiovascular Medical and Research Center: Rajaie Cardiovascular Medical and Research Center, IRAN, ISLAMIC REPUBLIC OF

## Abstract

**Objective:**

Increased nuchal translucency (NT; ≥ 2.5 mm) is closely associated with various chromosomal abnormalities, structural abnormalities, and genetic diseases. However, research on pathogenic copy number variations (CNVs) affecting pregnancy outcomes in fetuses with increased NT remains limited. This study aimed to summarize the clinical features, chromosomal abnormalities and obstetric outcomes of increased NT to provide a clinical reference for prenatal diagnosis.

**Methods:**

A retrospective analysis was conducted on 180 pregnant women with increased NT who underwent invasive prenatal testing via conventional karyotyping and single nucleotide polymorphism (SNP) array analysis between January 2021 and December 2025. These cases were selected from a cohort of 1,678 pregnant women.

**Results:**

Chromosomal karyotyping revealed a total of 24 chromosomal abnormalities. Chromosomal polymorphisms were also observed in four cases, including two cases with pericentric inversion of chromosome 9 [inv(9)(p12q13)], one case with 1qh+, and one case with 21pss (double satellites on the short arm of chromosome 21). In addition, SNP-array analysis detected CNVs in 13 fetuses with normal karyotypes. Among the 24 fetuses harboring pathogenic variants, 21 (87.5%) exhibited aneuploidy. These aneuploid cases included 16 fetuses with trisomy 21 syndrome and 5 fetuses with either trisomy 18 syndrome or Klinefelter syndrome, with trisomy 21 accounting for 76.2% (16/21) of the aneuploid cohort. Among the CNVs detected by SNP-array, one was classified as pathogenic, one as likely pathogenic*,* and the remaining as variants of uncertain significance (VUS). Postnatal follow-up of 10 infants with VUS revealed growth and speech developmental delays in two cases, while no obvious abnormalities were observed in the remaining eight.

**Conclusions:**

Aneuploidy was found to be the predominant type of abnormality in fetuses with increased NT. This study suggests that high-resolution SNP-array exhibit greater sensitivity in detecting chromosomal microstructural abnormalities and can serve as an effective complement to conventional karyotyping.

## Introduction

In the early 1990s, increased nuchal translucency (NT) was first identified as a sonographic marker for fetal chromosomal abnormalities in the first trimester, particularly for the detection of Down syndrome (trisomy 21) [1]. Fetal NT measurement is a clinically significant parameter assessed by ultrasound between 11 and 13^+6^ weeks of gestation. Currently, an NT thickness of ≥3.5 mm, corresponding to the 99th percentile irrespective of gestational age and fetal crown-rump length (CRL), is recognized as one of the most sensitive indicators of fetal chromosomal abnormalities and is a definitive indication for invasive prenatal diagnosis [2]. Fetuses with increased NT have an elevated risk of chromosomal aneuploidy, pathogenic copy number variations (pCNVs), prenatal or perinatal death, and other adverse pregnancy outcomes [3–5]. Moreover, the risk of fetal abnormalities and adverse outcomes is proportional to the degree of NT enlargement. When increased NT is detected in the first trimester, invasive prenatal diagnostic techniques-such as chorionic villus sampling (at 11–13^+6^ weeks) or amniocentesis (typically performed at approximately 20 weeks of gestation) are often employed to exclude chromosomal defects. Recent studies have reported that increased NT is associated with not only chromosomal aneuploidy but also chromosomal CNVs, cardiac and other structural anomalies, and single-gene disorders (e.g., Roberts syndrome and *KAT6A* syndrome) [4–7]. In addition to prenatal screening, NT measurement and maternal age are independent indicators in first trimester [5].A cohort analysis and literature review found that fetuses with an NT of 3.0–3.4 mm have a high risk of 1:7.4 for a chromosomal aberration, of which 69% involve chromosomes 13, 18, and 21 [8]. However, the cutoff value for defining increased NT as an indication for invasive prenatal diagnosis remains inconsistent and varies between countries. For example, fixed cutoff values such as ≥2.5 mm, ≥ 3.0 mm, and ≥3.5 mm are used in different national guidelines [9]. These inconsistent criteria have led to controversial data and analyses, thereby limiting the clinical utility of NT measurement in prenatal diagnosis. In accordance with clinical practice, the present study adopted a cutoff value of 2.5 mm to define NT thickening [5,9].

For many years, standard karyotyping has been considered the “gold standard” for detecting chromosomal abnormalities in prenatal diagnosis. This technique can identify numerical abnormalities and balanced translocations–such as free trisomies, Robertsonian translocations, and large structural aberrations (>5–10 Mb)–but it requires prolonged cell culture and offers limited resolution for cytogenetic detection [10,11]. In contrast, SNP-array enables the detection of CNVs across the whole genome. This method can identify chromosomal imbalances, including aneuploidy and unbalanced rearrangements, with a particular advantage for detecting microdeletions and microduplications that cannot be resolved by standard karyotyping [12,13].

Therefore, we conducted a retrospective analysis to summarize the clinical features, chromosomal abnormalities, and obstetric outcomes associated with increased NT in 180 fetuses, aiming to provide a clinical reference for prenatal diagnosis.

## Materials and methods

### Patient data

**Fig 1:**
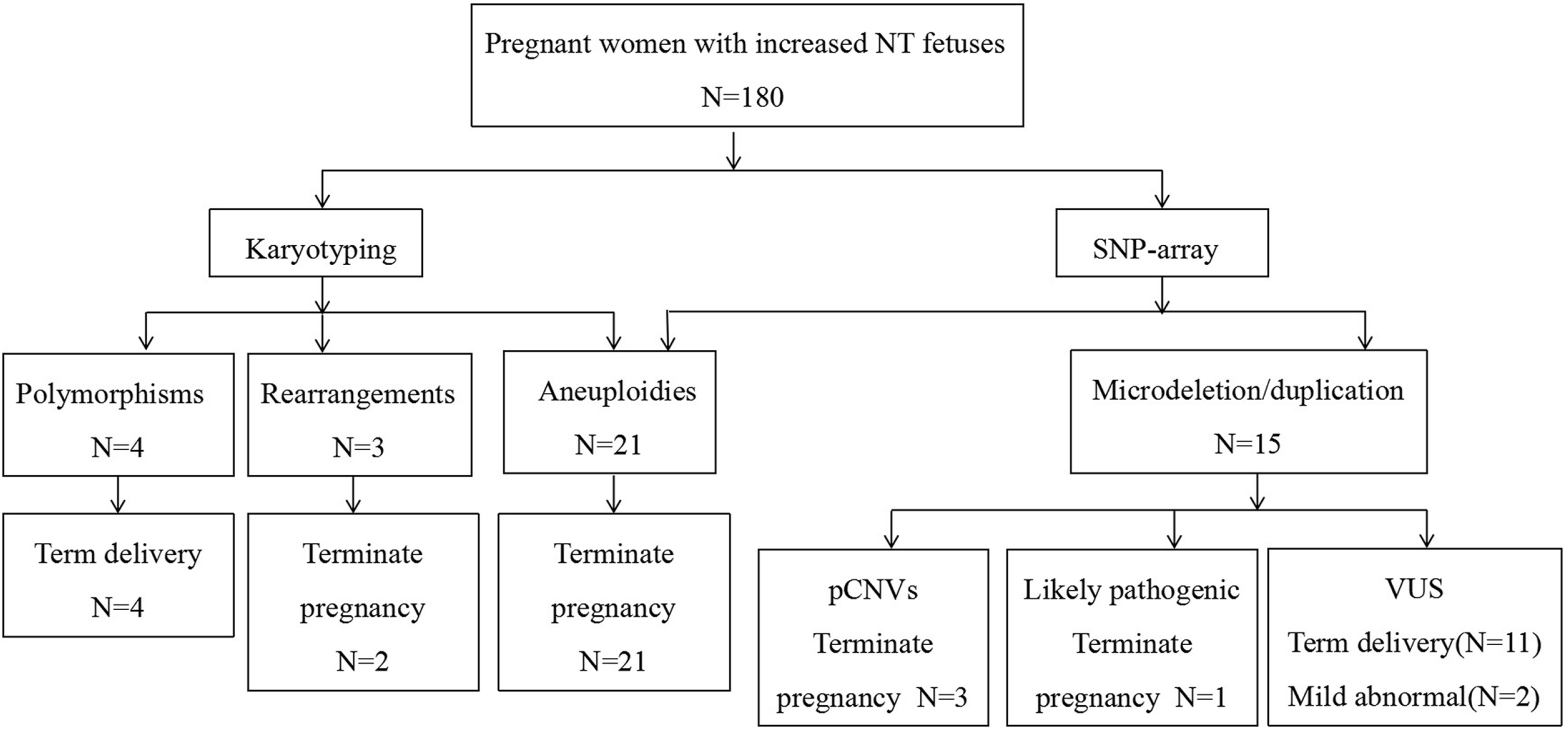
Flowchart of clinical pathways for 180 fetuses with increased NT. Karyotyping revealed 28 cases of polymorphisms, rearrangements, and aneuploidies. SNP-array detected 15 additional microdeletions/duplications, including 3 pathogenic, 1 likely pathogenic, and 11 VUS CNVs, with most VUS cases showing favorable outcomes.

The dates we accessed the data for research purposes were 12/05/2023. 180 pregnant women with increased NT, who accepted invasive prenatal diagnosis for traditional karyotyping and SNP array analysis were retrospectively selected from January 2021 to December 2025 in the diagnostic institutions of the Ningde Municipal Hospital. Fetal samples were collected from foetus with increased NT at a median gestational age of 19^+5^ weeks (range: 18^+1^–25^+4^ weeks) using amniocentesis. The median age at pregnancy was 30 years (range: 18–43 years). Under ultrasound guidance, 30 mL amniotic fluid was extracted from all 180 pregnant women, of which 20 mL was used for chromosome karyotyping by culturing in two lines and the remaining 6–10 mL was used for SNP array analysis (Fig 1). All pregnant women underwent genetic counselling and signed informed consent forms prior to the invasive diagnostic procedures. Once pathogenic CNVs in the foetus were confirmed, approximately 2.0 mL peripheral venous blood was collected from the parents to verify inheritance. DNA was extracted using the Gentra Puregene Blood Kit (QIAGEN, Santa Clara, CA, USA). This study was approved by the Ethics Committee of Ningde Normal University (NSYKYLL-2023-028).

### Cytogenetic analysis

Lymphocytes and amniocytes were analysed by traditional karyotyping using the Giemsa banding technique at 400–550 band resolution.

### CMA (SNP array)

According to the manufacturer’s instructions, the QIAamp® DNA Blood kits (Qiagen, Valencia, CA, USA) was used to directly extract fetal genomic DNA from uncultured amniotic fluid. DNA was quantified using a NanoDrop spectrophotometer (Thermo Fisher Scientific, Waltham, MA, USA) according to the manufacturer’s instructions. Briefly, fetal DNA was digested, attached to adapters, amplified, purified, fragmented, biotin-labelled, and hybridised using an Affymetrix® CytoscanTM 750 K array system (Affymetrix, Santa Clara, CA, USA) that contains SNP and CNV probes and detects CNVs, mosaics (mosaic proportion >10%), and loss of heterozygosity (LOH). The SNP array detected imbalances within the kilobase range. The results obtained from the CytoScan arrays were analysed using Chromosome Analysis Suite software (Affymetrix) with annotations of the genome version GRCH37 (hg19) [10]. The detected copy number gains or losses were systematically evaluated by comparing the values available in publicly available databases, including the Database of Genomic Variants (http://projects.tcag.ca/variation/), the DECIPHER database (http://decipher.sanger.ac=/), International Standards for Cytogenomic Arrays (https://www.iscaconsortium.org/), and Online Mendelian Inheritance in Man (OMIM; http://www.omim.org). CNVs were categorised as benign, likely pathogenic, pathogenic, or VOUS according to American College of Medical Genetics guidelines [14,15]. SNP array analysis of DNA from maternal and paternal blood samples was performed to determine whether the CNVs detected in the fetal samples were de novo or inherited. All de novo CNVs were validated using metaphase fluorescence in situ hybridisation (FISH).

### Statistical analysis

IBM SPSS Statistics version 20 (IBM Corp., Armonk, NY, USA) was used for the statistical analysis. The chi-square (χ^2^) test was performed to determine the significance of the differences between the NT values. Differences were considered statistically significant at *P* < 0.05.

## Results

A total of 180 pregnant women carrying fetuses with NT measurements ≥2.5 mm, with a median maternal age of 30 years (range: 18–43 years), were enrolled in this study. Based on the degree of NT thickening, the participants were categorized into four groups: 2.5–2.9 mm, 3.0–3.4 mm, 3.5–3.9 mm, and ≥4.0 mm. The detection rates of aneuploidy in these groups were 12.8%, 15.4%, 5.1%, and 20.5%, respectively, while the rates of other abnormal copy number variants (CNVs) were 18.5%, 10.3%, 0%, and 5.1%, respectively. The highest aneuploidy detection rate was observed in fetuses with NT ≥ 4.0 mm (20.5%), whereas the highest rate of other abnormal CNVs occurred in the 2.5–2.9 mm group (18.5%). Notably, no chromosomal rearrangements or abnormal CNVs were detected in fetuses with NT measurements of 3.5–3.9 mm,although this finding may be attributable to the small sample size in this subgroup. Moreover, the prevalence of fetal aneuploidy was significantly higher in the group with NT ≥ 4.0 mm (38.1%; 8/21) than in the group with NT measuring 2.5–2.9 mm (23.8%; 5/21) (*χ*^*2*^ = 6.04, *P* = 0.014). All results are summarized in Table 1.

**Table 1:** Abnormal fetal karyotyping and copy number variations grouped by increased NT (11–13^+6^ weeks).

Group	Number of samples, n (%)	Age (mean± SDs)	Number of abnormal karyotyping and CNVs		
			Aneuploidy, n (%)	Rearrangements, n (%)	Abnormal CNVs, n (%)
NT 2.5–2.9 mm	66 (36.7)	30.26 ± 5.1	5 (12.8)	1 (2.6)	7 (18.0)
NT 3.0–3.4 mm	64 (35.5)	29.05 ± 3.5	6 (15.4)	2 (5.1)	4 (10.3)
NT 3.5–3.9 mm	19 (10.6)	30.3 ± 4.6	2 (5.1)	0	0
NT ≥ 4.0 mm	31 (17.2)	30.4 ± 5.5	8 (20.5)	2 (5.1)	2 (5.1)

NT: Nuchal translucency; chromosomal rearrangements including duplications, inversions, and balanced translocations**.**

### Cytogenetic analysis of fetuses with increased NT

Karyotyping and SNP-array analysis identified 23 pathogenic chromosomal abnormalities among the 180 fetuses with increased NT. These included 21 pathogenic aneuploidies: trisomy 18 (Edwards syndrome, n = 4), trisomy 21 (Down syndrome, n = 16), and Klinefelter syndrome (47,XXY, n = 1). In addition, three fetuses harbored structural chromosomal abnormalities, comprising one case of 15q duplication syndrome, one case with a balanced reciprocal translocation t(4;13)(q31.1;q32), and one case with a derivative chromosome der(11)t(11;13)(q24;q31). Furthermore, chromosomal polymorphisms were observed in four fetuses, including two with pericentric inversion of chromosome 9 [inv(9)(p12q13)], one with increased heterochromatin on the long arm of chromosome 1 (1qh+), and one with double satellites on the short arm of chromosome 21 (21pss) as detailed in Table 2.

**Table 2:** Abnormal karyotyping results of fetuses with increased NT.

Case	Karyotype	SNP-array results	Pathogenicity classification	Postnatal outcome
1	47,XY,+18	arr[hg19](18)x3	P (Edward syndrome)	TP
2	47,XY,+18	arr[hg19](18)x3	P (Edward syndrome)	TP
3	47,XX,+18	arr[hg19](18)x3	P (Edward syndrome)	TP
4	47,XY,+18	arr[hg19](18)x3	P (Edward syndrome)	TP
5	47,XX,+21	arr[hg19](21)x3	P(Down syndrome)	TP
6	47,XX,+21	arr[hg19](21)x3	P(Down syndrome)	TP
7	47,XX,+21	arr[hg19](21)x3	P(Down syndrome)	TP
8	47,XY,+21	arr[hg19](21)x3	P(Down syndrome)	TP
9	47,XX,+21	arr[hg19](21)x3	P(Down syndrome)	TP
10	47,XX,+21	arr[hg19](21)x3	P(Down syndrome)	TP
11	47,XX,+21	arr[hg19](21)x3	P(Down syndrome)	TP
12	47,XX,+21	arr[hg19](21)x3	P(Down syndrome)	TP
13	47,XX,+21	arr[hg19](21)x3	P(Down syndrome)	TP
14	47,XX,+21	arr[hg19](21)x3	P(Down syndrome)	TP
15	47,XY,+21	arr[hg19](21)x3	P(Down syndrome)	TP
16	47,XX,+21	arr[hg19](21)x3	P(Down syndrome)	TP
17	47,XY,+21	arr[hg19](21)x3	P(Down syndrome)	TP
18	47,XY,+21	arr[hg19](21)x3	P(Down syndrome)	TP
19	47,XX,+21	arr[hg19](21)x3	P(Down syndrome)	TP
20	47,XX,+21	arr[hg19](21)x3	P(Down syndrome)	TP
21	47,XXY	arr[hg19](X)x2,(Y)x1	Klinefelter syndrome	TP
22	46,XX,dup(15)(q11.2q13.1)	arr[hg19]15q11.2q13.1(23,286,423-28,526,905)x3	P(15q duplication syndrome)	TP
23	46,XX,der(11)t(11;13)(q24;q31)	arr[hg19]11q24.2q25(127,752,401-134,937,416)x1 arr[hg19]13q31.1q34(87,674,122-115,107,733)x3	P	TP
24	46,XY,t(4;13)(q31.1;q32)	arr[hg19](1–22) × 2,(XX) × 1	Benign	2 years old, normal
25	46,XY,inv(9)(p12q13)	arr[hg19](1–22) × 2,(XX) × 1	Benign	6 months old,normal
26	46,XY,inv(9)(p12q13)	arr[hg19](1–22) × 2,(XX) × 1	Benign	8 months old,normal
27	46,XY,1qh+	arr[hg19](1–22) × 2,(XX) × 1	Benign	1.5 years old,normal
28	46,XX,21pss	arr[hg19](1–22) × 2,(XY) × 1	Benign	1 years old, normal

SNP, single nucleotide polymorphism; TP, termination of pregnancy; P, pathogenic.

### SNP array analysis results

SNP array analysis was consistent with karyotyping results in all 23 cases with chromosomal abnormalities. Moreover, SNP array analysis detected additional abnormal CNVs in 13 fetuses that had normal karyotypes. Among these 13 cases, 11 were classified as VUS, one as pathogenic, and one as likely pathogenic. The size of the abnormal CNVs ranged from 0.16 to 11.7 Mb. Of these, 12 were microdeletions or microduplications, and one involved a region of loss of heterozygosity (LOH) at 6p22.3p21.31. Detailed information is provided in Table 3.

**Table 3:** SNP array analysis of fetuses with increased NT in normal karyotyping.

Case	SNP-array results	Size (Mb)	Inheritance	Pathogenicity classification	Postnatal outcome
1	arr[GRCh37] 22q11.21(18,970,562_21,461,017)x3	2.4	*Refused*	Pathogenic	TP
2	arr[GRCh37]16p13.11p12.3(15359140-18172468)x3	2.8	*Refused*	Likely pathogenic	TP
3	arr[GRCh37]2q21.1(130,874,614-131,244,620)x1 arr[GRCh37]15q11.2(22,770,422-23,277,436)x1	0.36 0.5	*Refused*	VUS	TP
4	arr[GRCh37]2p12(78,631,710-79,995,493)x3	1.3	*Paternal*	VUS	1.6 years old,normal
5	arr[GRCh37]21q22.12q22.13(37,223,669-38,330,604)x3	1.1	*De novo*	VUS	2 years old, normal
6	arr[GRCh37]6p24.3p24.2(10,494,273-10,839,759)x1	0.38	*De novo*	VUS	4 years old, develop- mental delay
7	arr[GRCh37]20q11.22(32941157_33110042)x1	0.16	*Refused*	VUS	1.8 years old,normal
8	arr[GRCh37]7q31.32(122,066,578-123,066,288)x3	0.98	*De novo*	VUS	3 years old, normal
9	arr[GRCh37]16p11.2(29428532-30350748)x1	0.9	*Maternal*	VUS	2.5 years old, normal
10	arr[GRCh37]1q43(237892391_238400938)x3	0.5	*Refused*	VUS	1.8years old,normal
11	arr[GRCh37]18q12.3(42,111,540-43,387,120)x3	1.2	*De novo*	VUS	3 years old, language delayed
12	arr[GRCh37]6p22.3p21.31(23,972,905-35,703,669)x2hmz	11.7	*De novo*	VUS	2 years old, normal
13	arr[GRCh37]Yq11.223q11.23(24,820,716-28,420,380)x0	3.5	*Refused*	VUS	1.5 years old, normal

SNP, single nucleotide polymorphism; TP, termination of pregnancy; hmz, loss of heterozygosity.

The average maternal age, NT thickness, and CRL for the 13 fetuses with aneuploidies were 33.7years (range: 21–42years), 3.88 mm (range: 2.6–7.9 mm), and 6.33cm (range: 5.2–7.7cm), respectively (Table 4).

**Table 4:** Maternal and fetal information of 21 fetuses with aneuploidy and increased NT.

Case	Maternal age (years)	NT (mm)	CRL (cm)	Aneuploidy	Gestational age (weeks)
A132	35	3.6	6.27	Trisomy 21	12^+5^
A287	37	5.3	5.5	Trisomy 21	12^+1^
A291	36	3.2	5.6	Trisomy 21	12^+1^
A384	42	2.9	6.9	Trisomy 21	13
A456	35	6.0	7.7	Trisomy 21	13^+6^
A510	41	4.3	6.85	Trisomy 21	13^+3^
A653	21	3.3	7.1	Trisomy 21	13^+2^
A705	25	4.2	7.5	Trisomy 21	13^+4^
A671	28	3.0	5.2	Trisomy 18	12
B181	33	3.4	7.51	Klinefelter syndrome	13^+4^
B183	37	6.2	6.0	Trisomy 21	12^+4^
B219	24	2.6	6.1	Trisomy 21	12^+4^
B311	29	3.1	6.1	Trisomy 21	12^+4^
B369	33	3.6	6.4	Trisomy 21	12^+6^
B459	39	2.5	6.0	Trisomy 21	12^+4^
B507	36	2.7	6.7	Trisomy 18	13
B538	28	3.3	6.4	Trisomy 21	12^+5^
B540	37	2.8	7.2	Trisomy 21	13 ^+ 3^
B641	42	5.2	4.9	Trisomy 18	11^+5^
B673	35	2.6	5.7	Trisomy 21	12^+2^
S202	35	7.9	5.4	Trisomy 18	12

NT: Nuchal translucency; CRL: Crown rump length.

### Pregnancy outcomes and clinical follow-up

We conducted postnatal follow-up for all180 fetuses with increased NT; complete developmental data were obtained for 171 cases (95.0%), with follow-up durations ranging from 6 months to 4 years after birth. The remaining 9 cases (5.0%) were lost to follow-up, all of which had negative SNP-array results at the time of initial testing. Among the 171 successfully followed cases, pregnancy termination occurred in 25 cases. Of the remaining 146 live-born infants, 144 were developmentally healthy at birth and during follow-up period.Adverse outcomes were identified in two cases: one infant presented with isolated growth retardation and one exhibited speech delay. Down syndrome was the most common chromosomal aneuploidy observed among fetuses with NT thickening (76.2%, 16/21). Furthermore, SNP array analysis demonstrated a significantly higher detection rate for clinically relevant chromosomal abnormalities compared with conventional karyotyping.

## Discussion

Molecular diagnostic techniques, such as CMA and CNV-seq, increase pCNV detection rate by 4–7% compared with standard karyotyping in patients with increased NT [16,17]. CMA is a high-resolution method for genome-wide detection and is recommended as the primary test for prenatal diagnosis [18,19]. This method can detect CNVs associated with chromosomal imbalances, including aneuploidy and unbalanced rearrangements, with a particular advantage for identifying microdeletions and microduplications [12]. In pregnancies with a normal karyotype, CMA can detect clinically significant microdeletions/microduplications in approximately 1% of structurally normal fetuses and in 6% of cases with structural anomalies [20]. CMA encompasses two principal platforms: microarray-based comparative genomic hybridization (aCGH) and single-nucleotide polymorphism (SNP) arrays. SNP-array analysis offers the additional capability of detecting uniparental disomy (UPD), loss of heterozygosity (LOH), and low-level mosaicism [21,22]. Because SNP arrays can identify low-level mosaicism, submicroscopic microdeletions/microduplications, LOH, and UPD, this technology has replaced standard karyotyping as the first-tier prenatal diagnostic test in some countries [23]. Accordingly, in 2016, the American College of Obstetricians and Gynecologists (ACOG) recommended CMA as a first-line prenatal diagnostic test for evaluating fetuses with one or more major structural abnormalities [22,24]. In the present study, we performed whole-genome scanning using an SNP array alongside routine karyotyping in 180 fetuses from women with increased NT. We aimed to characterize the chromosomal abnormalities and CNVs in this cohort to explore the clinical value of applying SNP-array analysis in pregnancies complicated by increased NT.

In fetuses with NT > 2.5 mm, the probability of aneuploidy increased with NT thickness: the detection rates were 7.6% (5/66), 9.4% (6/64), 10.5% (2/19), and 25.8% (8/31) in the 2.5–2.9 mm, 3.0–3.4 mm, 3.5–3.9 mm, and ≥4.0 mm groups, respectively. Notably, in the group with NT ≥ 4.0 mm, more than one-quarter of the fetuses were diagnosed with chromosomal aneuploidy.

In this study, standard karyotyping of amniotic fluid from 180 pregnant women with fetuses having increased NT detected 21 fetuses with chromosomal aneuploidies, 3 with structural abnormalities, and 4 with chromosomal polymorphisms. SNP-array analysis additionally detected 13 fetuses with abnormal CNVs in those with normal karyotyping results. Among these, three pregnant women chose to terminate their pregnancies after genetic counseling, as they were unwilling to bear the potential risk of disease, because their fetuses carried pathogenic or likely pathogenic CNVs or VUS CNVs.

**Fetus 1** carried a 2.4Mb duplication at 22q11.21, which contained 46 protein-coding genes including *TBX1*. This segment involves the 22q11.2 recurrent (DGS/VCFS) region (proximal, A-D) (includes *TBX1*), 22q11.2 recurrent (DGS/VCFS) region (proximal, A-B) (includes *TBX1*)). According to the ClinGen database, there is sufficient evidence to support the pathogenicity of a threefold dose-sensitivity effect in these regions (Triplosensitivity score: 3). The disease associated with the triple dose-sensitive effect in this region is 22q11.2 duplication syndrome, and the clinical phenotypes include varying degrees of intellectual disability, learning disability, growth retardation, and hypotonia.

**Fetus 2** carried a 2.8Mb duplication at 16p13.11p12.3, which contained 12 RefSeq protein-coding genes including *MYH11*. This fragment contained the 16p13.11 recurrent region. In the ClinGen database, the triple dose sensitivity Score (TS) of the 16p13.11 recurrent region is 2. It has been reported that the duplication of 16p13.11 region is associated with different degrees of intellectual disability, developmental delay, autism spectrum disorder and other clinical phenotypes. The 16p13.11 duplication is mostly inherited from parents without abnormal phenotype, and the penetrance is approximately 8.4% [25,26]. The parents of Fetus 2 chose to terminate the pregnancy and declined verification of inheritance.

**Fetus 3** carried a 0.36 Mb microdeletion at 2q21.1 and a 0.5 Mb microdeletion at 15q11.2, which contained *CCDC115* and *SMPD4*, respectively. The ClinGen database did not show haplotype dose sensitivity for genes contained in this fragment. Therefore, the clinical significance of these deletions remains unclear. She did not accept the risk of uncertainty and decided to terminate the pregnancy at 23^+3^ weeks.

In our cohort, Down syndrome (trisomy 21) accounted for the highest proportion of aneuploidies (76.2%) among fetuses with increased NT. It has been reported that combined screening using maternal age, serological markers, and NT measurement can detect 80–90% of Down syndrome cases in the first trimester [5]. Furthermore, the diagnostic yield of CMA was approximately 6% for fetuses with structural anomalies and 49% for those with ultrasound abnormalities following positive serum screening results [20]. Our findings further confirm that increased NT is a high-risk factor for autosomal aneuploidy and is closely associated with CNVs, including microduplications, microdeletions, and LOH. Although VUS CNVs were detected in a small number of additional cases, only a few were associated with adverse pregnancy outcomes, which is consistent with previous studies [8].

In this cohort, SNP-array analysis in addition to conventional karyotyping provided an incremental diagnostic yield of 1.2% for pathogenic/likely pathogenic CNVs among fetuses with a normal karyotype. This yield is lower than the 1.9%–4.7% additional diagnostic rate reported in previous studies [27,28]. The discrepancy may be attributable to differences in sample size or population characteristic.

Nevertheless, this study has several limitations. First, the relatively small sample size may affect the accuracy and generalizability of the conclusions. Second, the focus on chromosomal abnormalities and CNVs alone limits our ability to assess potential associations between NT thickening and monogenic disorders. Third, long-term follow-up data on neurodevelopmental and clinical outcomes are still lacking. Future studies should include larger cohorts and more comprehensive genetic analyses (such as exome sequencing) to further elucidate these relationships.

## Conclusions

Increased NT is a major risk indicator for aneuploidy as well as pathogenic or likely pathogenic CNVs. Therefore, a clear understanding of the prevalence and spectrum of aneuploidies and CNVs associated with NT thickening can inform effective intervention strategies and contribute to improved prenatal counseling and pregnancy outcomes.

In this study, we identified aneuploidies and characterized CNVs—including microduplications, microdeletions, and LOH—in fetuses with increased NT. A notable proportion of these findings were classified as VUS, many of which were associated with favorable pregnancy outcomes during postnatal follow-up. Aneuploidy remains the primary factor influencing adverse fetal outcomes, particularly in fetuses with severe NT enlargement (NT ≥ 4.0 mm). However, NT thickening does not invariably lead to adverse outcomes or pregnancy loss. Comprehensive genetic evaluation and individualized counseling are essential for accurate risk assessment and optimal management of pregnancies with increased NT.
